# Identification of selective inhibitors for diffuse-type gastric cancer cells by screening of annotated compounds in preclinical models

**DOI:** 10.1038/s41416-018-0008-y

**Published:** 2018-03-12

**Authors:** Shu Shimada, Yoshimitsu Akiyama, Kaoru Mogushi, Mari Ishigami-Yuasa, Hiroyuki Kagechika, Hiromi Nagasaki, Hiroshi Fukamachi, Yasuhito Yuasa, Shinji Tanaka

**Affiliations:** 1 Department of Molecular Oncology, Graduate School of Medicine, Tokyo Medical and Dental University, Tokyo, Japan; 2 Center for Genomic and Regenerative Medicine, Juntendo University School of Medicine, Tokyo, Japan; 3 Chemical Biology Screening Center, and Department of Organic and Medicinal Chemistry, Institute of Biomaterials and Bioengineering, Tokyo Medical and Dental University, Tokyo, Japan; 4 Department of Hepato-Biliary-Pancreatic Surgery, Graduate School of Medicine, Tokyo Medical and Dental University, Tokyo, Japan

**Keywords:** Gastric cancer, Cancer screening

## Abstract

**Background:**

Diffuse-type gastric cancer (DGC) exhibits rapid disease progression and poor patient prognosis. We have previously established an E-cadherin/p53 double conditional knockout (DCKO) mouse line as the first genetically engineered one, which morphologically and molecularly recapitulates human DGC. In this study, we explored low-molecular-weight drugs selectively eliminating mouse and human DGC cells.

**Methods:**

We derived mouse gastric cancer (GC) cell lines from DGC of the DCKO mice demonstrating enhanced tumourigenic activity in immunodeficient mice and acquired tolerance to cytotoxic anti-cancer agents.

**Results:**

We performed a synthetic lethal screening of 1535 annotated chemical compounds, and identified 27 candidates selectively killing the GC cell lines. The most potent drug mestranol, an oestrogen derivative, and other oestrogen receptor modulators specifically attenuated cell viability of the GC cell lines by inducing apoptosis preceded by DNA damage. Moreover, mestranol could significantly suppress tumour growth of the GC cells subcutaneously transplanted into nude mice, consistent with longer survival time in the female DCKO mice than in the male. Expectedly, human E-cadherin-mutant and -low gastric cancer cells showed higher susceptibility to oestrogen drugs in contrast to E-cadherin-intact ones in vitro and in vivo.

**Conclusions:**

These findings may lead to the development of novel therapeutic strategies targeting DGC.

## INTRODUCTION

Gastric cancer (GC) is estimated as the third leading cause of cancer-related death in the world.^1^ GC is histologically classified into two major subtypes, intestinal-type and diffuse-type. Diffuse-type gastric cancer (DGC) in particular demonstrates infiltrative growth, and occasionally metastases to lymph nodes, resulting in worse prognosis.^2^ Although several clinical trials of chemotherapeutic drugs for advanced GC have been launched, overall survival rates have not been dramatically improved, approximately 20% in 5 years.^3–5^ Germline mutations of *CDH1* are frequently identified in hereditary DGC, while *TP53*, *CDH1* and *RHOA* mutations in sporadic DGC, but molecular mechanisms underlying diffuse-type gastric carcinogenesis have not been completely clarified.^6, 7^

We have recently established a mouse model of DGC, in which E-cadherin (*Cdh1*) and p53 (*Trp53*) are inactivated specifically in gastric mucosae.^8^ The penetrance is 100% for gastric neoplasm, contributing to the unfavourable mortality of 50% within a year. Poorly-differentiated and signet-ring cell adenocarcinoma cells are mainly distributed from mucosal to serosal layers in these mice. High frequency of lymph node dissemination and tumourigenicity in nude mice indicates the enhanced malignancy. Gene expression profiles of mouse DGC resemble those of human DGC, and mesenchymal markers and epithelial-mesenchymal transition (EMT)-regulators are over-expressed in mouse DGC as previously noted in human DGC. Taken together, the E-cadherin/p53 double conditional knockout (DCKO) mouse line is the first genetically engineered one which morphologically and molecularly recapitulates human DGC.^8^

An in vitro system is required to further extend the mouse model-based research, and we therefore derived GC cell lines harbouring biological and molecular traits closely similar to those in vivo from DGC of the DCKO mice. The powerful platform of the cell lines and model mice could facilitate the drug development and preclinical testing for DGC treatment. In this study, considering the poor understanding of targets in E-cadherin-deficient DGC and the easy availability of agents in clinical practice, we performed synthetic lethal screening of a library of well-characterised compounds by using these cell lines, and evaluated the selective cytotoxicity to mouse and human E-cadherin-deficient DGC in vitro and in vivo.

## MATERIALS AND METHODS

### Animal experiments, cell cultures, antibodies, and chemical compounds

The DCKO mice, *Atp4b-Cre*^*+*^*;Cdh1*^*loxP/loxP*^*;Trp53*^*loxP/loxP*^ genotype, were established as previously reported.^8^ The KSN nude mice were purchased from Charles River Laboratories Japan (Yokohama, Japan). All mouse procedures were approved by the Institutional Animal Care and Use Committee of Tokyo Medical and Dental University. Mouse GC cell lines were generated as described below. Mice bearing tumours were sacrificed, and the primary tumours were isolated. Small pieces were immediately minced from them under sterile conditions, decolonised at 4 °C overnight in DMEM/F12 media (Wako, Osaka, Japan) containing 10% fetal bovine sera (FBS), 100 U/ml penicillin, and 100 μg/ml streptomycin (Invitrogen, Carlsbad, CA), and subcutaneously injected into the male KSN nude mice. According to the same protocols, the transplanted tumour was dissected into aliquots which were explanted on the collagen-coated plates, and cultured in the DMEM/F12 media. The MDGC4SC1, 6 and 7 cell lines were subcloned from the MDGC4 by limiting dilution in DMEM (Wako) + 10% FBS. Similarly, the MDGC7, 8 and 9 cell lines were generated from the primary cancer (MDGC7 and 8) and lymph node dissemination (MDGC9) in F12 (Wako) supplemented with 5% horse or bovine sera (BS). The GIF7, 9 and 13 cell lines have previously reported,^9^ and maintained in DMEM + 10% FBS. Six HGC cell lines (MKN74, MKN7, MKN45, KATOIII, AGS and HSC58) were obtained as follows; MKN74, MKN7, MKN45 and KATOIII were purchased from RIKEN Cell Bank (Tsukuba, Japan); AGS was purchased from American Type Culture Collection (Manassas, VA); HSC58 was provided from Dr. Yanagihara (National Cancer Research Center, Tokyo, Japan). The HGC cells were cultured in RPMI 1640 (Wako) + 10% FBS. All cell lines were maintained in a humidified incubator at 37 °C in 5% CO_2_, and collected with 0.05% trypsin—0.02% EDTA solution (Wako). The antibodies and chemical compounds used in this study are enumerated in Supplementary Tables 1 and 2, respectively.

### Cell proliferation and viability assays

Cells were seeded at a density of 2 × 10^4^ cells per well in 12-well plates, and incubated overnight before each assay. The number of cell lines was estimated by using MTT in accordance with the manufacturer’s instructions. Briefly, 4 h after 100 μl of fresh media and 100 μl of 10 mg/ml MTT solutions (Dojindo, Kumamoto, Japan) were added to each well, the supernatant was discard, and the precipitate of formazan was dissolved in 500 μl of dimethyl sulfoxide (DMSO). The absorbance of the solution was measured on a microplate reader (Bio-Rad Laboratories, Hercules, CA) at 570 nm with background subtraction at 630 nm. Cell viability was calculated as the percentage of the number of cells treated with a drug to that with DMSO.

### Cell migration assay

Cells were seeded in 6-well plates at a density that was expected to reach 90–100% confluent as a monolayer after 24 h of growth. A scratch was made through the centre of each well by using a 1000 μl pipette tip, and the dislodged cells were removed by three washes. The adherent cells were incubated in the culture media containing 1% sera, and the gap distance of the wound was microscopically measured by time course.

### Sphere-forming assay

After harvested and passed through cell strainers (BD Biosciences, San Jose, CA), cells were seeded at a density of 1 × 10^3^ cells per well in 24-well Ultra-Low Attachment Plates (Corning, Corning, NY). Ten days after grown in serum-free media supplemented with epidermal growth factor (EGF), hydrocortisone and insulin, tumour spheres were microscopically quantified.

### Tumourigenicity in immunocompromised mice

Cells were suspended in 100 μl Matrigel (BD Biosceiences) and subcutaneously injected into the male KSN nude mice. Frequency of tumourigenic cells and *P*-value was calculated by using extreme limiting dilution analysis.^10^ The volume of the growing tumours was monitored once a week, and calculated by the formula; volume = length × width^2^ × 0.5. Chemical agents were treated after tumour became palpable (about 500 mm^3^). Tumour transplantation and drug administration were performed following the condition and schedule described in Supplementary Table 3.

### Drug screening

Cells were seeded in 100 μl of media including 1 × 10^3^ cells into 96-well plates. A compound library composed of 1535 well-characterised and off-patent compounds was generously provided from Chemical Biology Screening Center of Tokyo Medical and Dental University (http://www.tmd.ac.jp/mri/SBS/cbsc/). At 12 h, 100 mM of diluted compound solution was transferred from the 96-well stock plates into the 96-well assay plates, resulting in 100 and 10 μM final concentration for all compounds. Forty eight hours after compound treatment, 10 μl of WST-8 Reagent (Dojindo) per well was added, and the absorbance was measured on a microplate reader (Bio-Rad Laboratories) at 450 nm with background subtraction at 630 nm at 4 h.

### Cell cycle analysis

Cells were plated in 6-cm dishes and grown overnight. Forty eight hours after drug treatment, the cells were harvested, washed with phosphate-buffered saline (PBS) and fixed with 70% ethanol overnight at 20 °C. After rinsed with PBS containing 3% bovine serum albumin (BSA), the cells were resuspended in PBS with 50 μg/ml PI solution (Sigma-Aldrich, St. Louis, MO) and 10 μg/ml RNase A (Sigma-Aldrich) for 30 min on ice. The stained cells were counted by a FACSCalibur flow cytometer (BD Biosciences).

### Apoptosis analysis

Cells were seeded in 6-cm dishes and incubated overnight. Twenty four hours after each drug was administered, the cells were collected, rinsed with binding buffer (10 mM HEPES, pH 7.4; 140 mM NaCl; 2.5 mM CaCl_2_), and incubated in 100 μl of binding buffer containing 5 μl of Annexin V-FITC Reagent (MBL International Corporation, Woburn, MA) for 30 min on the ice. The labelled cells were sorted by a FACSCalibur flow cytometer (BD Biosciences). For caspase inhibition assays, cells were seeded at a density of 2 × 10^4^ cells per well in 12-well plates, and incubated overnight. Forty eight hours after treatment of 20 μM z-VAD-FMK (MedChem Express, Monmouth Junction, NJ) and 100 μM mestranol, cell viability was calculated by using MTT as described above.

### RNA extraction and RT-PCR

Total RNA was isolated from cells with TRIzol Reagent (Invitrogen). Contaminated DNA was removed by digestion with RNase-free DNase using DNA-*free* Kit (Applied Biosystems, Carlsbad, CA). For single-stranded complementary DNA synthesis, 1 μg of total RNA was reverse-transcribed by SuperScript III Reverse Transcriptase (Invitrogen). The primer sets and amplification conditions for PCR are listed in Supplementary Table 4. Glyceraldehyde-3-phosphate dehydrogenase (Gapdh) RNA was used as endogenous controls.

### Protein extraction and Western blotting

Cells were lysed using RIPA Buffer (Thermo Fisher Scientific, Waltham, MA) with a protease inhibitor cocktail kit (Sigma-Aldrich). Aliquots containing 30 μg of cell lysates were denatured in 5× Sample Buffer (Wako), electrophoretically resolved on SDS-polyacrylamide gels (Wako), and then transferred onto Immobilon polyvinyldifluoride membranes (Millipore, Billerica, MA). The membrane blots were blocked with 2% skimmed milk (Cell Signaling Technology, Danvers, MA) for an hour at room temperature, and then incubated with primary antibodies at 4 °C overnight. After the appropriate secondary antibodies were added for an hour, the signals were developed with Immun-Start AP Substrate (Bio-Rad) and observed by using LAS-3000 (Fujifilm, Tokyo, Japan).

### Immunocytochemistry

Cells were seeded onto small coverslips in 6-well plates, and incubated for 24 h to allow cell attachment. The cells were fixed with 4% paraformaldehyde at 4 °C for 15 min, permeabilised with 0.1% Triton X-100 for five minutes prior to incubation in 3% BSA for 30 min at room temperature. The blocking buffer was removed, and the cells were incubated with primary antibodies at 4 °C for an hour. After washed with PBS, they were additionally incubated with fluorescence-conjugated secondary antibodies for an hour, and the cellular DNA was subsequently counterstained by ProLong Gold Antifade Reagent with 4’,6-diamidino-2-phenylindole (DAPI) purchased from Invitrogen. The slides were viewed with a fluorescent microscope (Carl Zeiss, Oberkochen, Germany).

### Microarray analysis

Integrity of obtained RNA was assessed by using the Agilent 2100 BioAnalyzer (Agilent Technologies, Palo Alto, CA). All samples were confirmed to have an RNA integrity number (RIN) greater than 7. Cyanine-3 (Cy3) labelled complementary RNA was prepared from total RNA for each sample by using the Low Input Quick Amp Labeling kit (Agilent). Hybridisation and signal detection of SurePrint G3 Mouse Gene Expression v2 8×60K Microarray Kit (G4852B, Agilent) was performed following the manufacturer’s protocols. Normalised signal intensities were acquired by Feature Extraction Software (Agilent) and then transformed into log 2 base. All the microarray datasets are deposited in GEO (GEO accession: GSE102297).

### Statistical analysis

Unsupervised clustering analysis based on Ward’s method and principal component analysis for 505 genes with interquartile range (IQR) values greater than 4.0 were performed by using the R statistical software (version 3.0.3). Statistical analysis was also conducted by using the R statistical software. In cell viability assays, each *P*-value was calculated with the GraphPad Prism 7 software (San Diego, CA). *P*-value less than 0.05 was considered statistically significant.

## RESULTS

### Establishment of mouse E-cadherin/p53-deficient DGC cell lines

**Fig. 1:**
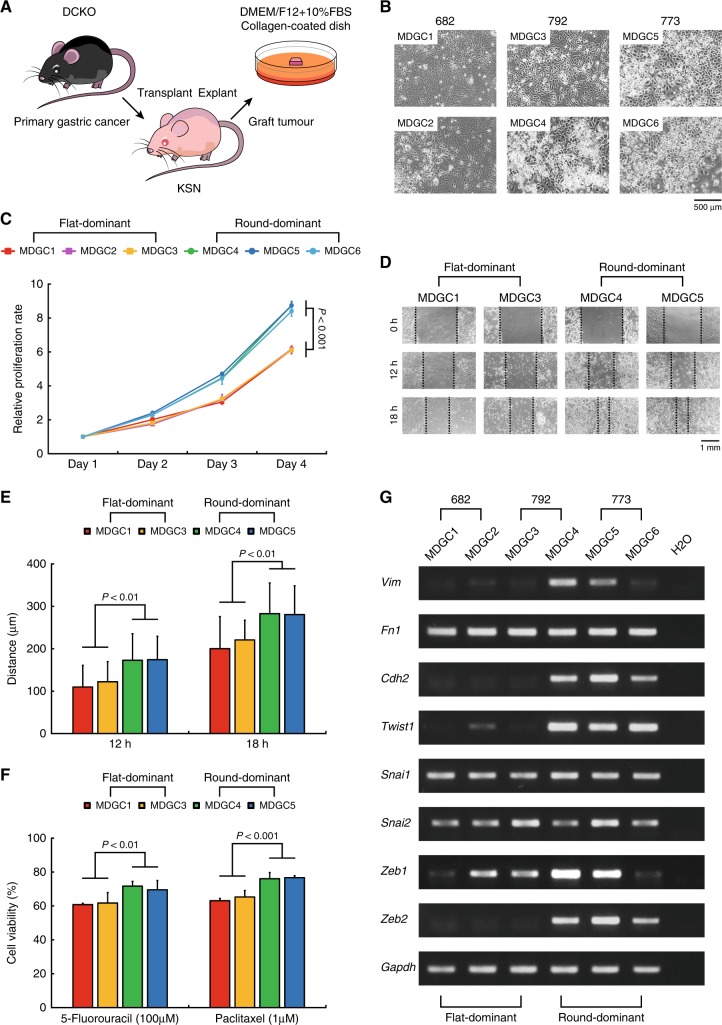
Establishment of two morphologically, biologically and molecularly distinct types of mouse DGC cell lines. **a** Schematic of culture of the MDGC1 to 6 cell lines established from DGC of the DCKO mice. **b** Representative phase-contrast images of the MDGC1 to 6 cell lines. **c** Proliferation curves of the flat-dominant and round-dominant MDGC cell lines. The value of each samples was relative to that at Day 1. Bars show standard deviations. *P*-value was calculated by analysis of variance (ANOVA) with Tukey-Kramer post hoc test. **d** Representative phase-contrast images of wound-healing. **e** Quantification of cell migration. Bars show standard deviations. *P*-value was calculated by ANOVA with Tukey-Kramer post hoc test. **f** Quantification of resistance against chemotherapeutic agents. Bars show standard deviations. *P*-value was calculated by ANOVA with Tukey-Kramer post hoc test. **g** Expression levels of mesenchymal markers and EMT-regulators examined by RT-PCR

We first took into culture three transplanted tumours (#682, 792 and 773) of DGC of the DCKO mice as schematically illustrated in Fig. 1a, and isolated two cell lines rapidly expanding from each tumour (Supplementary Figure 1A). After cultured for a few passages, these six cell lines showed morphological heterogeneity, flat and round; the #682 and #773 tumours induced two flat-dominant (MDGC1 and 2) and round-dominant cell lines (MDGC5 and 6), respectively, whereas the #792 did one flat-dominant (MDGC3) and one round-dominant (MDGC4) as presented in Fig. 1b. We also confirmed complete recombination of the *Cdh1* and *Trp53* loci by performing genomic PCR in all of the six MDGC cell lines (Supplementary Figure 1B).

We next investigated biological and molecular characteristics of these two types of cell lines. Cell proliferation assays (Fig. 1c) and wound-healing assays (Fig. 1d,e) displayed that the round-dominant cell lines exhibited greater mitogenic and motile properties than the flat-dominant ones. The round-dominant cell lines were more refractory than the flat-dominant to two cytotoxic drugs commonly used for treatment of patients with advanced GC, 5-fluorouracil and paclitaxel (Fig. 1f). Similarly to the gene expression signatures of primary DGC of the DCKO mice, the expression levels of mesenchymal markers (*Vim* and *Cdh2*) and EMT-regulators (*Twist1* and *Zeb2*) were higher in the round-dominant cell lines than those in the flat-dominant cell lines (Fig. 1g). These findings suggested that mouse E-cadherin/p53-deficient DGC might consist of two subtypes of cancer cells with morphologically, biologically and molecularly distinct features.

### Evaluation of mouse E-cadherin/p53-deficienct DGC cell lines

**Fig. 2:**
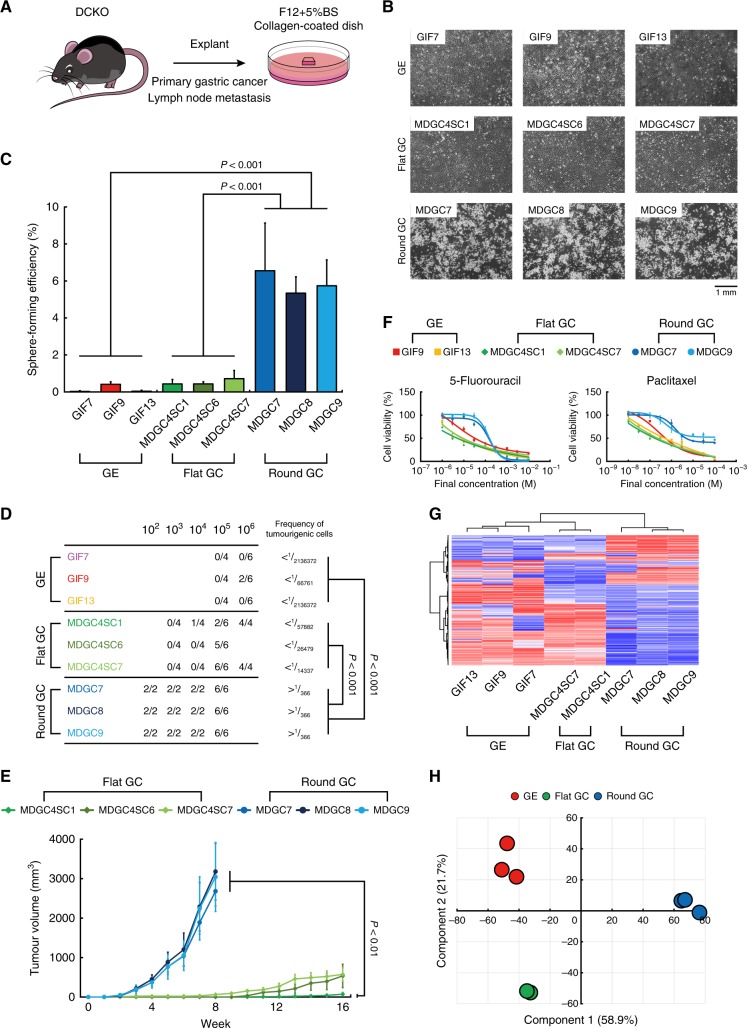
Evaluation of cancer stem cell-like properties of mouse DGC cell lines. **a** Schematic of culture of the MDGC7 to 9 cell lines established from DGC of the DCKO mice. **b** Representative phase-contrast images of the GE, flat and round GC cell lines. **c** Quantification of sphere-forming efficiency. Bars show standard deviations. *P*-value was calculated by ANOVA with Tukey-Kramer post hoc test. **d** Table of tumour-forming ability. **e** Tumour-growth curves of the flat and round GC cell lines. Bars show standard errors. *P*-value was calculated by ANOVA with Tukey-Kramer post hoc test. **f** Dose-response curves of two cytotoxic anti-cancer agents. Bars show standard deviations. **g** Unsupervised clustering analysis and (**h**) principal component analysis of the GE, flat and round GC cell lines

We succeeded in obtaining three subclones (MDGC4SC1, 6 and 7) composed of only flat cancer cells by limiting dilution, but it was difficult to maintain ones retaining the round phenotype for long periods. We then optimised culture conditions including sera, media and substrates, and newly established three cancer cell lines (MDGC7, 8 and 9) with the round shape from primary GC and lymph node metastasis of DGC of the DCKO mice (Fig. 2a), which harboured complete recombination of the *Cdh1* and *Trp53* loci as expected (Supplementary Figure 1B). We also possess cell lines derived from stomach mucosae of fetal p53-null mice.^9^

We now compared the mouse flat and round *Cdh1*^*−/−*^*;Trp53*^*−/−*^ GC cell lines with the mouse *Trp53*^*−/−*^ gastric epithelial (GE) cell lines as control (Fig. 2b). We first cultured these three types of cell lines with serum-free media in ultra-low attachment culture dishes, an in vitro measurement of tumourigenic activity. The round GC cell lines showed a 30-fold increase in sphere-forming capacities relative to the flat GC and GE (Fig. 2c, Supplementary Figure 2A). We next directly assessed the tumourigenic abilities by subcutaneously injecting them into nude mice. Tumours were generated with only 100 cells of the round GC, which were 100-fold and 1000-fold less than were required for tumour seeding by the flat GC and GE cells, respectively (Fig. 2d). Tumours of the round GC cell lines grew much more rapidly than those of the flat GC (Fig. 2e). In transplanted tumours derived from the round GC cells, poorly differentiated cancer cells and signet ring carcinoma cells were abundantly distributed, and gland-like structures formed by moderately differentiated cancer cells were also scattered (Supplementary Figure 2B). In contrast, the flat GC cells generated tumours mainly composed of the gland-like structures, indicating that the round GC cells could maintain CSC-like properties and be at the higher level of tumour hierarchy than the flat GC cells. A strong resistance to conventional chemotherapeutic agents is also an important aspect of malignancy. Indeed, 5-fluorouracil and paclitaxel decreased the cell number of the round GC cell lines less than that of the flat GC and GE (Fig. 2f). As predicted from these in vitro results, when intraperitoneally administered into nude mice bearing palpable tumours of the MDGC7 cell line, 5-fluorouracil (50 mg/kg/week) failed to reduce tumour sizes (Supplementary Figure 2C). Unsupervised clustering analysis (Fig. 2g) and principle component analysis (Fig. 2h) of gene expression profiles could clearly distinguish these three cell types from each other, although the round and flat GC cells shared the similar genetic backgrounds. Among 505 differentially expressed genes, 142 genes were up-regulated in the round GC cell lines compared with the GE and flat GC included *Twist1*,^11^ consistent with the above-mentioned data (Fig. 1g). Taken together, we established bona fide cancer cell lines of DGC of the DCKO mice.

### First and second screenings of a library of known chemical compounds

**Fig. 3:**
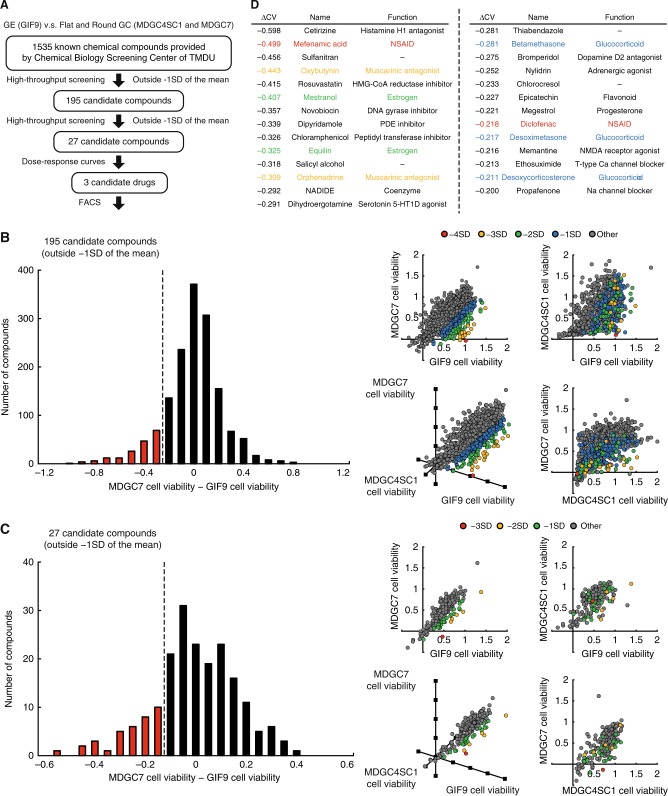
Screening of a library of well-characterised compounds by using mouse E-cadherin/p53-deficient DGC cells. **a** Overview of the proof-of-concept assay to identify drugs selectively killing the round GC cell lines. **b** Histogram (left panel) and two-dimensional and three-dimensional scatter plots (right panel) of the difference between the MDGC7 and GIF9 cell viability against 1535 test compounds. **c** Histogram (left panel) and two-dimensional and three-dimensional scatter plots (right panel) of the difference between the MDGC7 and GIF9 cell viability against 195 candidate compounds. **d** List of 27 candidates extracted by the first and second screening, which intriguingly contained some classes of drugs clinically used (as the same colour). ∆CV means the difference between the GIF9 and MDGC7 cell viability

We speculated that drugs selectively killing the GC cell lines might be a silver bullet toward E-cadherin-deficient DGC. On the basis of this reasoning, we designed a proof-of-concept assay to identify such drugs (Fig. 3a), namely, we screened 1535 well-characterised compounds provided from Chemical Biology Screening Center of Tokyo Medical and Dental University by using the GE (GIF9), flat GC (MDGC4SC1) and round GC (MDGC7) cell lines. Before starting the ATP-based cell viability assays on 96-well plates, we examined the range where the cell number was proportionally correlated with the absorbance (Supplementary Figure 3A), and determined that seeding 1000 cells per well is appropriate for this assay. A screening window coefficient, Z’-factor, is requested to qualify a screening assay.^12^ Under exposure of high concentration (5 mM) of 5-fluorouracil as positive control, Z’-factors of the cell lines were calculated as approximately 0.5, assuring the reliability of this screening system (Supplementary Figure 3B).

We first screened 1535 test compounds including conventional chemotherapeutic drugs on this platform (Fig. 3b), and highlighted the difference between the GIF9 and MDGC7 cell viability (∆CV) in a histogram (Supplementary Table 6). We identified 195 chemical compounds outside −1 standard deviation (SD) of the mean of ∆CV as candidates with selective toxicity toward the round GC cell lines. We next reevaluated the 195 hit compounds in duplicate, and created a histogram of ∆CV and a scatter plot (Fig. 3c). In the same way, 27 candidates outside −1 SD of the mean of ∆CV were enumerated in the table of Fig. 3d, which intriguingly contained some classes of clinically available drugs; mefenamic acid and diclofenac (red) are classified as non-steroidal anti-inflammatory drugs (NSAIDs), prophylactic use of which contributes to lower relative risks of gastrointestinal cancer; ^13^ oxybutynin and orphenadine (orange) are muscarinic antagonists widely prescribed for patients with benign prostatic hyperplasia, and Zhao and colleagues have recently documented that physiological and pharmacological inhibition of muscarinic acetylcholine receptors suppresses gastric tumourigenesis;^14^ mestranol and equilin have oestrogen-like structure and function, consistent with the potential role of oestrogen in explaining the male predominance of stomach cancer;^15^ betamethasone, desoxymetasone and desoxycorticosterone belong to the glucocorticoid class, and are generally used for palliative care of terminal cancer patients. These similarity could assist the accuracy of our scheme.

### Third and fourth screenings of a library of known chemical compounds

**Fig. 4:**
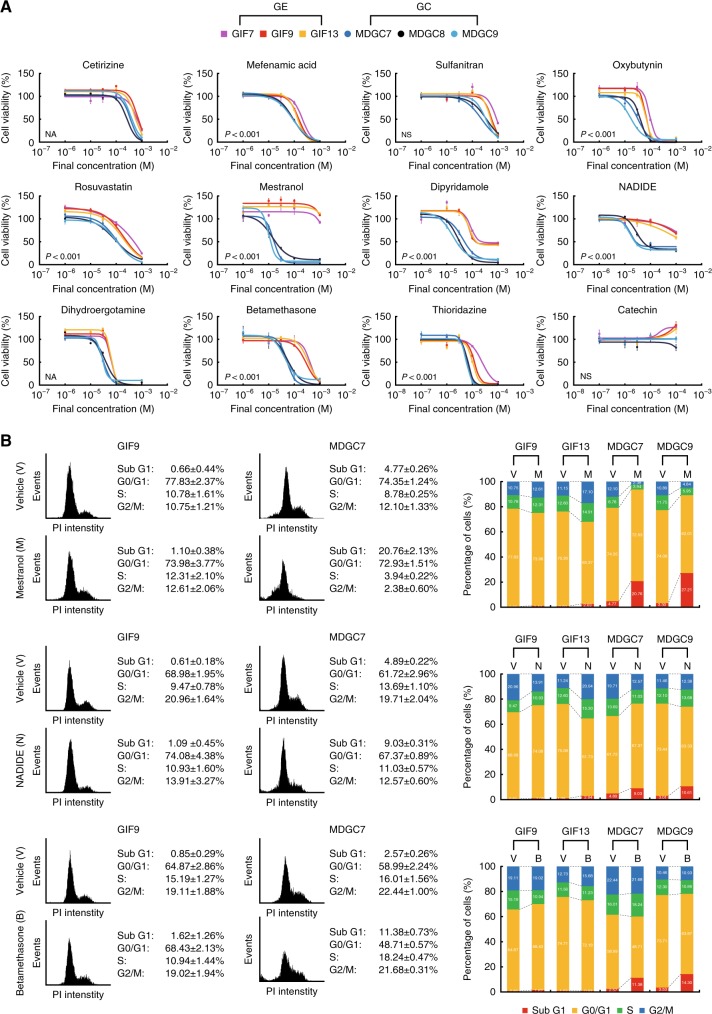
Identification of candidate compounds selectively killing mouse E-cadherin/p53-deficient DGC cells. **a** Dose-response curves of ten chemical compounds selected from the 27 candidate compounds in addition to thioridazine and catechin against the GE and round GC cell lines. Bars show standard deviations. *P*-value was calculated from the ANOVA table. NA not applicable., NS not significant. **b** Flow cytometric analysis with PI staining. The left and right panels show the representative histograms and percentage graphs of cells in each cell cycle, respectively

For further studies, after excluding antibiotics and antiseptics, we selected ten drugs and two alternates, thioridazine (a dopamine D2 receptor antagonist, ∆CV = −0.121) and catechin (an isomer of epicatechin, not included in the compound library), from the 27 candidate substrates listed in Fig. 3d in addition to four drugs, quercetin, salinomycin, flutamide and bicalutamide. Since thioridazine,^16^ quercetin^17^ and salinomycin^18^ have been detected as drugs targeting cancer stem cells (CSCs) by screening of annotated compound libraries, we hypothesised that they could also selectively kill the round GC cell lines harbouring CSC-like properties (Fig. 2). Garnett and collaborators have screened a panel of several hundred cancer cell lines with 130 drugs under clinical and preclinical investigation,^19^ and their public data suggest that somatic mutations of *CDH1* gene are associated with cellular responses to an androgen receptor antagonist bicalutamide (*P* = 6.87 × 10^−3^), categorised in the same class as flutamide (∆CV = −0.271). Thus, we examined their effects across a range of doses, and confirmed that all of them provided evidence of selective toxicity toward the round GC cell lines (Fig. 4a, Supplementary Figure 4A). These results were not only supportive for the reliability of this screening assay, but also consistent with the previous reports of the drugs targeting CSCs^16–18^ and E-cadherin-mutant cancer cells.^19^ We compared drug sensitivity among three types of cell lines, the GE, flat and round GC, and found that the 12 candidate drugs were subdivided into two groups, ones selectively killing both the flat and round GC cell lines (e.g., mefenamic acid, rosuvastatin, dipyridamole and NADIDE) and ones doing only the round GC (Supplementary Figure 4B). Among the candidates, mestranol, NADIDE and betamethasone in particular had broad therapeutic windows between the GE and GC cell lines. We then performed flow cytometric analysis with propidium iodide (PI) staining with two types of cell lines to determine cell cycle profiles and apoptotic events. Treatment with mestranol, NADIDE and betamethasone increased the sub-G1 population only in the GC cell lines, consistent with the results of dose-response curves (Fig. 4b).

### Effects of oestrogen on mouse E-cadherin-deficient GC

We thus focused on mestranol, the most potent of the 1535 test compounds through the four steps of the screenings, and one of oestrogen derivatives frequently used for hormone replacement therapy. Observing that the 27 candidate substrates extracted by the second screening contained two oestrogen drugs (Fig. 3d), we retrospectively reanalysed the results of the first screening (Supplementary Table 6), and elucidated that the 195 hit compounds outside −1 SD of the mean of ∆CV included six compounds with oestrogen-like function such as 17β-oestradiol and tamoxifen, while the remainder did twelve, implying the class effect of oestrogen on cell viability of the GC cell lines (*P* = 0.0193, Fisher’s exact test). This hypothesis was encouraged by the correlation between E-cadherin mutation and sensitivity of androgen receptor antagonists in this (Supplementary Figure 4A) and the previous comprehensive drug screenings.^19^

**Fig. 5:**
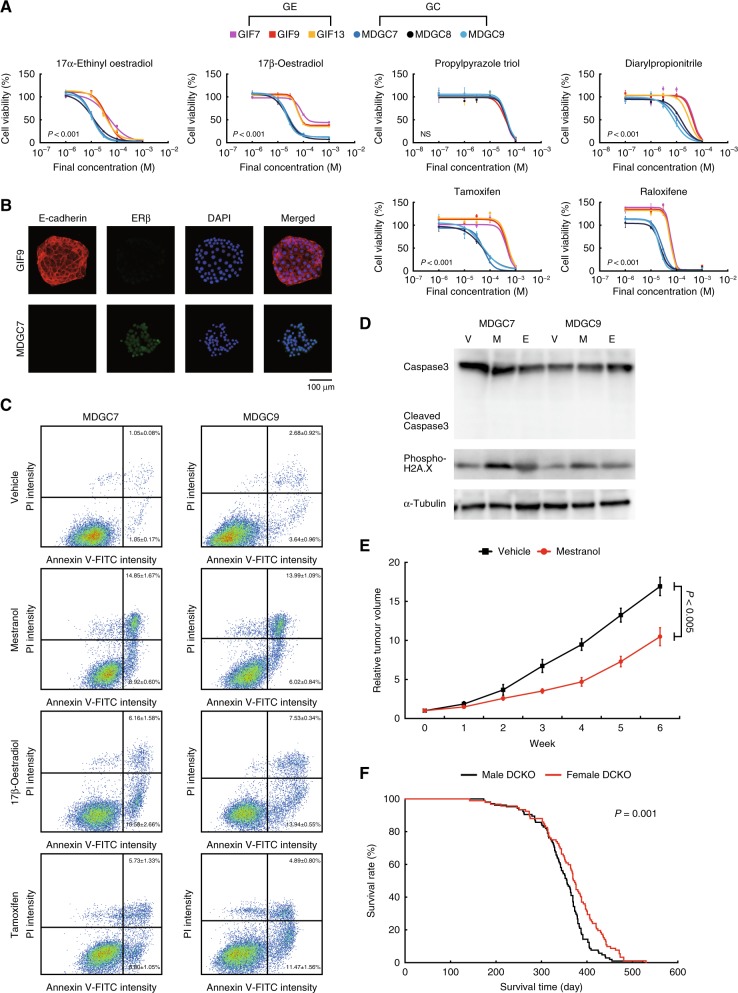
Effects of oestrogen drugs on mouse E-cadherin/p53-deficient DGC cells. **a** Dose-response curves of oestrogen drugs against the GE and round GC cell lines. Bars show standard deviations. *P*-value was calculated from the ANOVA table. NS not significant. **b** Representative fluorescence microscopy images of the GE and round GC cells stained with antibodies against E-cadherin (red) and ERβ (green). Nuclei were counterstained with DAPI (blue). **c** Flow cytometric analysis with Annexin V-FITC and PI costaining. **d** Immunoblots of caspase 3 and phosphorylated H2A.X after treatment with vehicle (V), mestranol (M) and 17β-oestradiol (E) in the round GC cell lines. **e** Tumour-growth curves of the MDGC7 cells in nude mice under treatment with mestranol (0.5 mg/kg/day, orally administered). Bars show standard errors. *P*-value was calculated by Welch’s *t*-test. **f** Kaplan–Meier curves of the male and female DCKO mice. The median survival time of the female (371.5 days, *n* = 92) was significantly longer than that of the male (356 days, *n* = 105). *P*-value was calculated by the log-rank test

We tried to explore the molecular mechanism and to identify oestrogen drugs with the most selective toxicity toward the GC cell lines (Fig. 5a, Supplementary Figure 5A). Exposure to not only 17α-ethinyl oestradiol (a metabolite of mestranol) and 17β- oestradiol but also tamoxifen and raloxifene (recently reclassified as selective oestrogen receptor modulators), abrogated cell viability of the round GC cell lines, similarly to the results of mestranol. Two homologues of oestrogen receptors (ERs), ERα and ERβ, have different distribution and function among normal tissues as well as neoplasms including stomach cancer.^15^ Propylpyrazole triol (ERα agonist) showed the similar cytotoxicity against the GE and GC cell lines, whereas diarylpropionitrile (ERβ agonist) specifically killed the GC, suggesting that ERβ could mainly mediate cell death of the GC cell lines. These data were supported by the higher expression levels of ERβ in the GC than those in the GE (Fig. 5b), and consistent with preventive effects of ERβ on digestive system carcinogenesis.^15^ Compared with normal gastric mucosae of *Atp4b-Cre*^−^*;Cdh1*^*loxP/loxP*^*;Trp53*^*loxP/loxP*^ mice, ERβ was overexpressed in DGC of the DCKO mice at the RNA level (Supplementary Figure 5B). Oestrogen-induced cell death occurs through an increase in proapoptotic genes ^20^ or DNA double-strand breaks.^21^ Mestranol, 17β-oestradiol and tamoxifen triggered cell apoptosis in the round GC cell lines by using flow cytometric analysis with Annexin V-fluorescein isothiocyanate (FITC) and PI costaining (Fig. 5c). These events were accompanied with a significant increase of phosphorylation of H2A.X, not cleavage of caspase 3 (Fig. 5d), and an irreversible caspase inhibitor z-VAD-FMK could not rescue the cytotoxicity of the GC cells (Supplementary Figure 5C). Taken together, oestrogen drugs could induce DNA damage specifically in the GC cell lines via ERβ, and mestranol was the best candidate among them in the end. We then orally administered mestranol (0.5 mg/kg/day) into nude mice with palpable inoculated tumours of the MDGC7 cells, and observed tumour-growth inhibition (Fig. 5e). The effects of mestranol on sphere-forming efficiency were compatible to those on cell proliferation in the round GC cells (Fig. 4a and Supplementary Figure 5D), indicating that mestranol could exhibit toxic activities, but not inhibit stem cell-like properties. Moreover, the median survival time of the female DCKO mice was significantly longer than that of the male (*P* = 0.001, the log-rank test), implying the tumour-suppressive roles of oestrogen in DGC of the DCKO mice (Fig. 5f).

### Effects of oestrogen on human E-cadherin-deficient GC cells

**Fig. 6:**
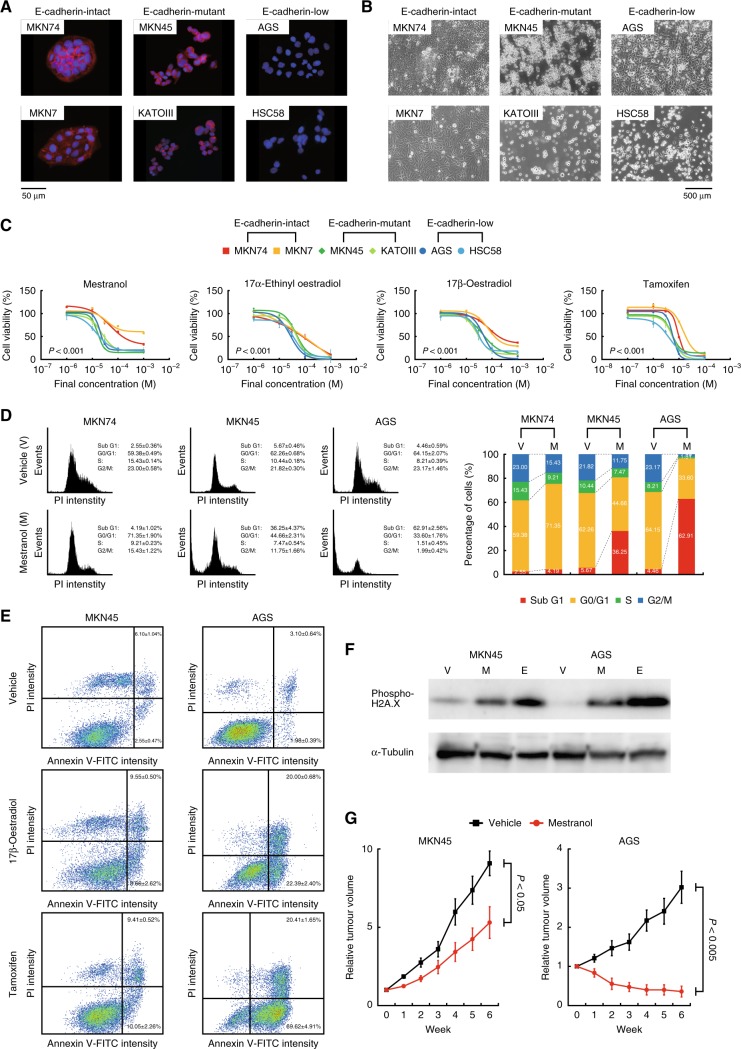
Effects of oestrogen drugs on human E-cadherin-deficient gastric cancer cells. **a** Representative fluorescence microscopy images of the HGC cells stained with antibodies against E-cadherin (red). Nuclei were counterstained with DAPI (blue). **b** Representative phase-contrast images. **c** Dose-response curves of oestrogen drugs against the HGC cell lines. Bars show standard deviations. *P*-value was calculated from the ANOVA table. NS not significant. **d** Flow cytometric analysis with PI staining. The left and right panels show the representative histograms and percentage graphs of cells in each cell cycle, respectively. **e** Flow cytometric analysis with Annexin V-FITC and PI costaining. **f** Immunoblots of phosphorylated H2A.X after treatment with vehicle (V), mestranol (M) and 17β-oestradiol (E) in the E-cadherin-mutant (MKN45) and -low (AGS) cells. **g** Tumour-growth curves of the E-cadherin-mutant (MKN45) and -low (AGS) cells in nude mice under treatment with mestranol (0.5 mg/kg/day, orally administered). Bars show standard errors. *P*-value was calculated by Welch’s *t*-test

We examined whether oestrogen drugs could exert therapeutic function for human E-cadherin-deficient GC. We initially assessed the distribution and expression of E-cadherin in several human gastric cancer (HGC) cell lines at the protein level (Fig. 6a, Supplementary Figure 6A) as well as the mutation status at the mRNA and DNA levels (Supplementary Figures S6B and C), and divided them into three groups; E-cadherin-intact (MKN74 and MKN7), E-cadherin-mutant (MKN45 and KATOIII) and E-cadherin-low (AGS and HSC58). Treatment with four drugs with oestrogen-like activity including mestranol selectively impaired cell viability of the E-cadherin-mutant and -low HGC cell lines (Fig. 6c). Mestranol triggered cell apoptosis, not cell cycle arrest, in the E-cadherin-deficient HGC cell lines similarly to 17β-oestradiol and tamoxifen, which was preceded by DNA damage (Fig. 6d-f, Supplementary Figures S6D-S6F). Mestranol administration suppressed transplanted tumour growth of the E-cadherin-mutant and E-cadherin-low cell lines, but not that of the E-cadherin-intact ones (Fig. 6g, Supplementary Figure 6G). Thus, cellular responses of E-cadherin-deficient GC cells to oestrogen could be highly conserved across two different species.

To investigate the relationship between gender, histological subtype, E-cadherin/p53 status and prognosis in clinical samples of GC, we used public data provided from the Cancer Genome Atlas Research Network (TCGA), which included 176 tumours (76 DGC and 100 IGC) with gene mutation, copy number alteration, DNA methylation, mRNA expression and pathological data. Surprisingly, the female DGC patients had better prognosis in overall survival than the male (*P* = 0.006, the log-rank test), although there was no difference between the female and male IGC patients (*P* = 0.725) as shown in Supplementary Figure 7A. Next, we divided the 176 clinical specimens of TCGA data set into the E-cadherin-deficient and -intact groups with and without somatic mutation, biallelic loss, promoter hypermethylation (*β*-value ≥ 0.5) or low expression (RSEM < 2000) of *CDH1* gene, respectively. In 76 DGC samples, 10 of 29 E-cadherin-deficient tumours harboured somatic mutation or biallelic loss of *TP53* gene, whereas 19 of 47 E-cadherin-intact tumours did, suggesting that E-cadherin/p53-deficiemt GC accounted for approximately 15% of DGC, and therefore was not rare (*P* = 0.636, Fisher’s exact test). Although not in the E-cadherin-intact group (*P* = 0.362, the log-rank test), overall survival rate of the female patients was higher than that of the male in the E-cadherin-deficient group (*P* = 0.048), which was consistent with the prognosis of the DCKO mice (Fig. 5f and Supplementary Figure 7B).

We performed Single Sample Gene Set Enrichment Analysis (ssGSEA) for tumour samples with high and low expression levels of *CDH1*, termed as CDH1-high and -low, with two gene sets associated with oestrogen signal transduction, ESTROGEN_RESPONSE_EARLY and ESTOGEN_RESPONSE_LATE. The ssGSEA scores of the CDH1-low group for both of the gene sets were significantly lower than those of the CDH1-high group (Supplementary Figure 7C), implying that only E-cadherin-deficient GC in which the oestrogen signal pathway was inactivated could survive.

## Discussion

The United States National Cancer Institute 60 human tumour cell lines anti-cancer drug screen (NCI60) emerged in the late 1980s as a powerful drug discovery tool.^22, 23^ However, cellular reactions in vitro are not able to reflect those in vivo due to two failings; cell lines have genetically, epigenetically and biologically changed under culture conditions; cell lines no longer maintain the tumour original properties present in the primary cancer. In an effort to address these shortcomings, patient-derived xenografts transplanted into immunodeficient rodents have recently been used for preclinical modelling. These models are appropriate for validating the in vivo effects of candidate drugs, but not for screening chemical libraries due to difficulty of primary culture. Since tumour genotype and epigenotype variation in tumour and between patients (intratumour and interpatient heterogeneity) is non-negligible, large-scale screenings are required for discovery of potent agents targeting the common molecular mechanism. We then solved these problems by a mouse model-based study, that is, establishing a genetically engineered mouse model recapitulating human cancer, deriving cell lines from the mouse tumour, performing a synthetic lethal screening by using the cell lines, and validating tumour suppressor activity of candidate drugs against human cancer. Early passage cells from cancer of a genetically engineered mouse model harbour the biological and molecular traits of the original tumours, and a few of them are sufficient for screening assays due to similar genetic background. In addition, by using the mice with carcinoma in situ, anti-cancer effects of the candidate drugs can be precisely evaluated on the platform mimicking tumour microenvironment including cancer-associated fibroblasts and immune cells.

Currently, most molecularly targeted drugs are inhibitors of driver oncogenes, because it appears more straightforward to repress a hyperactivated oncogene than to restore the function of inactivated tumour suppressor genes.^24^ Among several promising strategies of drug development for cancer with mutations in tumour suppressor genes, we here applied a comprehensive approach for E-cadherin-deficient DGC, that is, a screening with a collection of well-established annotated compounds. This process of finding new uses outside the scope of the original medical indication for existing drugs is known as repositioning,^25^ and offers two significant advantages over conventional de novo drug discovery and development; firstly, from the molecular function of compounds selectively killing cancer cells, the addicted signal pathway could be predicted; secondly, safer and shorter routes to the clinic are possible because in vitro and in vivo screenings, lead optimisation and chemical toxicology have already been completed. Excellent examples of this concept “repositioning” are thioridazine and salinomycin. Thioridazine, a Food and Drug Administration (FDA)-approved antipsychotic dopamine receptor antagonist, was reprofiled as an anti-CSC drug from libraries of known compounds, and demonstrated that dopamine D2 receptor antagonism could account for the loss of stemness.^16^ Since Gupta et al. initially did, several other groups have noted that salinomycin attenuates CSC-like properties in various types of cancer, and identified that this potassium ionophore overcomes ABC transporter-mediated multidrug resistance and inhibits oxidative phosphorylation in mitochondria on which CSCs mainly rely more than on glycolysis. Our screening assay with 1535 well-characterised compounds also revealed that differentially expressed ERβ could be a potential target for E-cadherin-deficient DGC, and provided convincing evidence of clinical use of oestrogen drugs including mestranol for prevention and treatment of this subtype of GC.

Our mouse model-based study could hint favourable prognosis of female patients with DGC. It sounds odd that oestrogen protects against the development of DGC, which is believed to be encountered in young female patients, although there is a strong and enigmatic male dominance in the incidence of GC with a male-to-female ratio of approximately 2:1.^15^ The cumulative risk of hereditary DGC by age 60 was estimated to be higher for females than for males, but not significantly.^26^ Patients with DGC younger than 40 years showed a nearly equal male-to-female ratio (1.3:1), whereas patients older than 40 showed a male preponderance (2.3:1) in a series of 66 GC probands for germline *CDH1* mutations.^27^ In a large-scale cohort study, the diffuse-type is more common in males than in females for nearly all age groups.^28^ Females had a significantly lower risk of dying compared to males in patients whose tumours were poorly differentiated.^29^ There is also mounting evidence for the clinical potency of exogenous oestrogen on GC. A seemingly protective effect of hormone replace therapy, such as mestranol treatment, on GC risk has been reported in several human studies from different populations.^15^ In animal models, administration of oestrogen to *N*-methyl-*N*’-nitro-nitrosoguanidine (MNNG)-treated rat^30^ and *Helicobacter pylori*-infected INS-GAS mice^31^ decreased their incidence of GC. Thus, an intrinsic tumour-suppressor role of oestrogen in E-cadherin-deficient DGC is assisted by these epidemiological and experimental findings.

Since tamoxifen administration in mice causes extensive parietal cell damage by active acid secretion through H,K-ATPase,^32^ it is possible that estrogen analogues are also toxic to the GC cells, which were originated from parietal cells of the DCKO (*Atp4b-Cre*^*+*^*;Cdh1*^*loxP/loxP*^*;Trp53*^*loxP/loxP*^) mice. In immunohistochemical analysis, cleaved caspase 3 was not detected in parietal cells of the DCKO mice orally treated with mestranol (0.5 mg/kg/day) for a week, suggesting few adverse effects of mestranol on parietal cells (Supplementary Figure 8A). Because RT-PCR analysis demonstrated that the expression levels of *Atp4a* and *Atp4b* encoding H,K-ATPase subunits were extremely lower in the GE and GC cell lines than those in normal gastric mucosae containing parietal cells (Supplementary Figure 8B), it did not seem that mestranol specifically eliminated the GC cells by inducing acid secretion through H,K-ATPase. The two evidences disproved the hypothesis above.

We here screened the compound library composed of off-patent drugs, and highlighted a single potent drug, mestranol, targeting E-cadherin-deficient DGC. It is also of interest to investigate how the other 26 candidates listed in Fig. 3d could exhibit the specific toxicity to this subtype of GC, and to perform screening with other libraries containing novel molecularly targeted agents by using this platform.

## Electronic supplementary material

Supplementary Information

Supplementary Figure 1

Supplementary Figure 2

Supplementary Figure 3

Supplementary Figure 4

Supplementary Figure 5

Supplementary Figure 6

Supplementary Figure 7

Supplementary Figure 8

Supplementary Tables
