# Supplementary material for: Identification of selective inhibitors for diffuse-type gastric cancer cells by screening of annotated compounds in preclinical models
Source: Br J Cancer. 2018 Mar 12;118(7):972–84. doi: 10.1038/s41416-018-0008-y (PMC5931092; doi:10.1038/s41416-018-0008-y)
Supplement: Supplementary file 1 — Supplementary Information [file 41416_2018_8_MOESM1_ESM.docx]

**Identification of selective inhibitors for diffuse-type gastric cancer cells by screening of annotated compounds in preclinical models**

Shu Shimada^1^, Yoshimitsu Akiyama^1^, Kaoru Mogushi^1,2^, Mari Ishigami-Yuasa^3^, Hiroyuki Kagechika^3^, Hiromi Nagasaki^1^, Hiroshi Fukamachi^1^, Yasuhito Yuasa^1^, Shinji Tanaka^1,4^

^1^Department of Molecular Oncology, Graduate School of Medicine, Tokyo Medical and Dental University, Tokyo, Japan. ^2^Center for Genomic and Regenerative Medicine, Juntendo University School of Medicine, Tokyo, Japan. ^3^Chemical Biology Screening Center, and Department of Organic and Medicinal Chemistry, Institute of Biomaterials and Bioengineering, Tokyo Medical and Dental University, Tokyo, Japan. ^4^Department of Hepato-Biliary-Pancreatic Surgery, Graduate School of Medicine, Tokyo Medical and Dental University, Tokyo, Japan.

**SUPPLEMENTARY MATERIALS AND METHODS**

**Immunohistochemistry.** Tissues were fixed overnight in 4% paraformaldehyde, embedded in paraffin, and sectioned (4 μm thick). Sections were immersed in sodium citrate (pH 6.0) buffer for antigen retrieval, and subsequently incubated with a primary antibody against cleaved caspase 3 (5A1E, 1:1000) purchased from Cell Signaling Technology (Danvers, MA) at 4 °C overnight. They were probed with anti-rabbit IgG antibody labelled with peroxidase Histofine Simple Stain MAX-PO (Nichirei Bioscience, Tokyo, Japan), and visualized with diaminobenzidine.

**DNA extraction.** Cell pellets were suspended in TNE Buffer (10 mM Tris-HCl, pH 8.0; 150 mM NaCl; 2 mM EDTA; 0.5% SDS) with 1 mg/ml proteinase K (Roche, Mannheim, Germany) at 48 °C for an hour. Genomic DNA was obtained from cells by phenol-chloroform extraction.

**Sequencing analysis.** To detect *CDH1* and *TP53* mutations in human gastric cancer cell lines, genomic DNA and cDNA were amplified with the primer pairs listed in supplementary table S5. The PCR products were sequenced by using the BigDye Terminator v3.1 Cycle Sequencing Kit on an Applied Biosystems 3730xl DNA analyzer (Applied Biosystems, Carlsbad, CA) according to the manufacturer’s instructions.

**Statistical analysis.** For comprehensively analysis of gastric cancer, a public data set provided from the Cancer Genome Atlas Research Network (TCGA) were obtained through the cBioPortal site (http://www.cbioportal.org/). Single Sample Gene Set Enrichment Analysis (ssGSEA) of gene expression data was performed with the MSigDB gene sets (H: hallmark gene sets; version 4.0) on the GenePattern site (http://software.broadinstitute.org/cancer/software/genepattern/) in accordance with the manufacturer’s instructions (Subramanian *et al*, 2005).

**SUPPLEMENTARY FIGURE LEGENDS**

**Supplementary Figure S1.** Primary culture of DGC of the DCKO mice. (**A**) Representative phase-contrast images of the primary culture of mouse DGC (#792). (**B**) Genotyping of the *Cdh1* and *Trp53* loci in mouse DGC cell lines (GC). Untruncated and truncated *Trp53* were amplified at intron 10 and exon 2-10 deletion mutant of *Trp53* gene. Untruncated and truncated *Cdh1* were also done at intron 10 and exon 5-10 deletion mutant of *Cdh1* gene. The primer sets were listed in our previous paper (Shimada *et al*, 2012). Since the mouse gastric epithelial cells (GE) were established from stomach mucosae of p53-null mice (*Trp53^Δ2-6/Δ2-6^*), the *Trp53* locus in them could be amplified by using the primer set targeting intron 10 of *Trp53* gene.

**Supplementary Figure S2.** Evaluation of cancer stem cell-like properties of mouse DGC cells. (**A**) Representative phase-contrast images of spheres composed of the GE, flat and round GC cell lines. (**B**) Representative images of hematoxylin and eosin staining of transplanted tumors derived from the flat and round GC cells. (**C**) Tumour-growth curves of the MDGC7 cell line in nude mice with treatment of 5-fluorouracil (50 mg/kg/week, intraperitoneally injected). Bars show standard errors. *P*-value was calculated by Welch's *t*-test. NS, not significant.

**Supplementary Figure S3.** Validation of screening system. (**A**) Standard curves derived from titration of the GE, flat and round GC cell lines. The standard curves indicate a liner response between the cell number (500, 1000, 2000 and 4000) and absorbance. (**B**) Z'-factor calculation. Cells treated with or without 5-fluorouracil (5 mM) were used as positive and negative controls, respectively. Solid and dotted lines show the mean and the mean ± 3 SD, respectively.

**Supplementary Figure S4.** Dose-response curves of candidate compounds by using mouse GE, flat and round GC cells. (**A**) Dose-response curves of two chemical compounds targeting CSCs (quercetin and salinomycin) and two androgen receptor antagonists (flutamide and bicalutamide) against the GE and round GC cell lines. (**B**) Dose-response curves of 10 chemical compounds selected from the 27 candidate compounds in addition to thioridazine and catechin against the GE, flat and round GC cell lines. Bars show standard deviations. Bars show standard deviations.

**Supplementary Figure S5.** Effects of oestrogen drugs on mouse GE, flat and round GC cells. (**A**) Dose-response curves of oestrogen drugs against the GE, flat and round GC cell lines. Bars show standard deviations. (**B**) Relative mRNA expression levels of *Esr2* in normal stomach tissues of *Atp4b-Cre^−^;Cdh1^loxP/loxP^;Trp53^loxP/loxP^* mice (*n* = 6) and DGC of the DCKO mice (*n* = 6). The value of each sample was relative to the mean value of normal stomach group. Bars show standard deviations. *P*-value was calculated by Welch's *t* test. (**C**) Quantification of cell viability of the GC cell lines pretreated with an irreversible caspase inhibitor z-VAD-FMK (20 μM) under exposure to mestranol (100 μM). Bars show standard deviations. *P*-value was calculated by ANOVA with Tukey-Kramer post hoc test. NS, not significant. (**D**) Quantification of sphere-forming efficiency under treatment with mestranol. Bars show standard deviations.

**Supplementary Figure S6.** Correlation between of E-cadherin status and oestrogen drug sensitivity of human gastric cancer cell lines. (**A**) Immunoblots of E-cadherin in HGC cell lines. (**B**) Sequencing analysis of *CDH1*. A 12-bp sequence is deleted at the DNA level (c.821_832del) and contributes to in-frame deletion (p.274_277del) in MKN45. A base substitution in the last nucleotide of exon 7 (c.G1008A) alters the splicing process, producing frameshift insertion of the subsequent intron sequences in KATOIII (p.336fs). These mutations of *CDH1* were consistent with the previous report (Oda *et al*, 1994). (**C**) Sequencing analysis of *TP53*. A nonsynonymous single nucleotide variant of HSC58 (c.T402G) causes the encoding of a dominant-negative mutant of p53 (p.F134L). (**D**) Flow cytometric analysis with PI staining. The left and right panels show the representative histograms and percentage graphs of cells in each cell cycle, respectively. (**E**) Flow cytometric analysis with Annexin V-FITC and PI costaining. (**F**) Immunoblots of phosphorylated H2A.X after treatment of vehicle (V), mestranol (M) and 17β-estradiol (E) in the E-cadherin-mutant (KATOIII) and -low (HSC58) cells. (**G**) Tumour-growth curves of the E-cadherin-intact (MKN74) and -low (HSC58) cells in nude with treatment of mestranol (0.5 mg/kg/day, orally administered). Bars show standard errors. *P*-value was calculated by Welch's *t*-test. NS, not significant.

**Supplementary Figure S7.** Relationship between gender, histological subtype, E-cadherin status and prognosis in TCGA data set of gastric cancer. (**A**) Kaplan-Meier curves of patients with IGC (male, *n* = 64; female, *n* = 36) and DGC (male, *n* = 49; female, *n* = 27). (**B**) Kaplan-Meier curves of patients with the E-cadherin-intact (male, *n* = 92; female, *n* = 47) and -deficient (male, *n* = 21; female, *n* = 16) gastric cancer. The E-cadherin-deficient group was composed of tumors with somatic mutation, biallelic loss, promoter hypermethylation or low expression of *CDH1* gene encoding E-cadherin. (**C**) Comparison of ssGSEA scores of oestrogen signal transduction between tumor samples with the top (CDH1-high, *n* = 44) and bottom (CDH1-low, *n* = 44) quartile expression of *CDH1*. Horizontal lines show median values. *P*-value was calculated by Mann-Whitney *U* test.

**Supplementary Figure S8.** Effects of mestranol on parietal cells. (**A**) Immunohistochemical staining of cleaved caspase 3. Serial sections of the samples were stained with hematoxylin and eosin. (**B**) RT-PCR analysis of *Atp4a* and *Atp4b* in the GE and GC cell lines. Two samples of mouse normal stomach were positive controls.

**REFERENCES**

Oda T, Kanai Y, Oyama T, Yoshiura K, Shimoyama Y, Birchmeier W, Sugimura T, Hirohashi S (1994) E-cadherin gene mutations in human gastric carcinoma cell lines. *Proc Natl Acad Sci USA* **91**(5): 1858–1862.

Subramanian A, Tamayo P, Mootha VK, Mukherjee S, Ebert BL, Gillette MA, Paulovich A, Pomeroy SL, Golub TR, Lander ES, Mesirov JP (2005) Gene set enrichment analysis: A knowledge-based approach for interpreting genome-wide expression profiles. *Proc Natl Acad Sci USA* **102**(43): 15545–15550.
